# Indiscriminate characterization of persons with disabilities: strictly nosographic legislation without functional assessment

**DOI:** 10.47626/1679-4435-2026-1543

**Published:** 2026-04-15

**Authors:** Gustavo de Almeida

**Affiliations:** 1 Occupational Health and Quality of Life Service, Federal Senate, Brasília, DF, Brazil

**Keywords:** disabled persons, disability evaluation, models, biopsychosocial, health policy, occupational health.

## Abstract

The concept of a person with a disability has evolved from a medical-biological perspective to a biopsychosocial understanding that considers the dynamic interaction between functioning, disability, and contextual factors, as proposed by the International Classification of Functioning, Disability and Health. However, a tension can be observed between this model and legislation that automatically recognizes certain conditions as disabilities without the corresponding functional assessment. Among these conditions are monocular vision, autism spectrum disorder, and fibromyalgia. This opinion article analyzes some legal, social, and ethical implications of this conceptual expansion in Brazil. Such expansion, by compromising the principles of positive discrimination, poses significant challenges to public policies guided by equity and distributive justice.

## INTRODUCTION

The concept of a person with a disability (PwD) has undergone significant transformations over the past decades, shifting from a predominantly medical-biological perspective to a broader biopsychosocial understanding. This change reflects the recognition that disability is not limited to the presence of a clinical condition but results from the interaction between individual impairments and social, environmental, and attitudinal barriers.

However, increasing tension can be observed between this model and legislative initiatives that promote the automatic recognition of certain health conditions as disabilities without the corresponding individualized functional assessment. This trend, sometimes driven by identity-based movements or by specific demands, may compromise normative coherence and affect the balance between equity and efficiency in the formulation of public policies.

In this essay, adopting a critical-analytical approach, bibliographic and documentary elements related to the recognition of PwD in Brazil are examined, with emphasis on three specific conditions: monocular vision, autism spectrum disorder (ASD), and fibromyalgia.

The objective is to discuss some legal, social, and ethical impacts resulting from the indiscriminate expansion of the concept of disability, as well as to reflect on the need for normative harmonization guided by the implementation of evaluative criteria that consider the diversity of individual experiences and the different degrees of functioning. In this way, the aim is to foster an essential debate on affirmative action policies aimed at PwDs.

## DEVELOPMENT

### NORMATIVE FRAMEWORKS

The criteria for recognizing PwD in Brazil reveal a scenario of contradictions resulting from the coexistence of different normative parameters.

Decree No. 3,298/1999, which for many years constituted the main federal regulation on the subject, reflects the medical-biological conception of disability, centered on diagnosis and predominant in the historical and cultural context in which it was drafted:

Art. 4. A person with a disability is considered to be one who falls into the following categories:

I - physical disability - complete or partial alteration of one or more segments of the human body, resulting in impairment of physical function, presenting in the form of paraplegia, paraparesis, monoplegia, monoparesis, tetraplegia, tetraparesis, triplegia, triparesis, hemiplegia, hemiparesis, amputation or absence of a limb, cerebral palsy, limbs with congenital or acquired deformity, except aesthetic deformities and those that do not produce difficulties in the performance of functions;

II - hearing disability - partial or total loss of auditory capacity, varying in degrees and levels as follows:

a) from 25 to 40 decibels (dB) - mild hearing loss;

b) from 41 to 55 dB - moderate hearing loss;

c) from 56 to 70 dB - marked hearing loss;

d) from 71 to 90 dB - severe hearing loss;

e) above 91 dB - profound hearing loss; and

f) anacusis;

III - visual disability - visual acuity equal to or less than 20/200 in the better eye, after the best correction, or a visual field smaller than 20º (Snellen chart), or the simultaneous occurrence of both situations;

I - physical disability - complete or partial alteration of one or more segments of the human body, resulting in impairment of physical function, presenting in the form of paraplegia, paraparesis, monoplegia, monoparesis, tetraplegia, tetraparesis, triplegia, triparesis, hemiplegia, hemiparesis, ostomy, amputation or absence of a limb, cerebral palsy, dwarfism, limbs with congenital or acquired deformity, except aesthetic deformities and those that do not produce difficulties in the performance of functions; (Wording given by Decree No. 5,296, 2004)

II - hearing disability - bilateral, partial or total loss of forty-one decibels (dB) or more, measured by audiogram at the frequencies of 500Hz, 1,000Hz, 2,000Hz and 3,000Hz; (Wording given by Decree No. 5,296, 2004)

III - visual disability - blindness, in which visual acuity is equal to or less than 0.05 in the better eye, with the best optical correction; low vision, defined as visual acuity between 0.3 and 0.05 in the better eye, with the best optical correction; cases in which the sum of the visual field measurement in both eyes is equal to or less than 60°; or the simultaneous occurrence of any of the previous conditions; (Wording given by Decree No. 5,296, 2004)

IV - intellectual disability - intellectual functioning significantly below average, with onset before the age of eighteen and limitations associated with two or more areas of adaptive skills, such as:

a) communication;

b) personal care;

c) social skills;

d) community use;

d) use of community resources; (Wording given by Decree No. 5,296, 2004)

e) health and safety;

f) academic skills;

g) leisure; and

h) work;

V - multiple disability - association of two or more disabilities [^1^].

However, in 2009, the Brazilian legal system incorporated the Convention on the Rights of Persons with Disabilities (CRPD), with constitutional amendment status. From this milestone onward, disability began to be understood from a biopsychosocial perspective, defined as the result of the interaction between impairments of a physical, mental, intellectual, or sensory nature and environmental barriers that restrict these individuals’ participation in society.

This change represents the overcoming of a strictly clinical perspective and the recognition of the parameters of the International Classification of Functioning, Disability and Health (ICF), of the World Health Organization (WHO), approved in 2001, as a complement to the International Classification of Diseases (ICD).

The paradigmatic shift was consolidated with the enactment of the Brazilian Inclusion Law of Persons with Disabilities (Law No. 13,146/2015), which explicitly adopts the social model of disability [^2^].

Despite this conceptual evolution, the proliferation of legislative proposals can be observed across the three levels of government that propose the automatic recognition of disability status for a wide range of conditions, including mental disorders, stuttering, keratoconus, rheumatoid arthritis, lupus, inflammatory bowel diseases, rare diseases, and retroviral seropositivity, etc. [^3^].

Among the most emblematic cases are monocular vision and ASD, both recognized as disabilities by federal law, as well as fibromyalgia, whose condition has been officially equated with disability in several Brazilian states and cities.

### FIBROMYALGIA

Fibromyalgia is a chronic pain syndrome of still not fully clarified etiology, characterized by increased sensitivity to pain [^4^]. Objective diagnostic confirmation is challenging, since there are no laboratory or imaging tests capable of establishing the diagnosis with certainty. Thus, its identification is essentially clinical and is based on the evaluation of symptoms reported by the patient, such as widespread chronic pain, fatigue, sleep disturbances, mood changes, and, in some cases, cognitive impairments [^5^].

Additionally, physical signs are often subtle or absent, which may generate diagnostic uncertainty, delays in recognizing the condition, and, in certain situations, the underestimation of complaints by health professionals themselves, particularly in forensic contexts.

However, fibromyalgia manifests heterogeneously among individuals. A widely known example is that of the singer Lady Gaga, who in 2017 canceled part of her tour due to pain associated with the disease. Subsequently, with therapeutic follow-up, pain management strategies, and lifestyle adaptations, the artist was able to resume her professional activities. She currently maintains an active career, which includes intense rehearsals and prolonged performances, demonstrating the possibility of living with the condition while maintaining satisfactory levels of productivity and quality of life [^6^].

Recently, Law No. 15,176/2025 [^7^] established the National Policy for the Protection of the Rights of Persons with Fibromyalgia, Complex Regional Pain Syndrome, and other related conditions [^8^]. Unlike some previous regulatory provisions at the state or municipal level, which did not establish distinctions regarding case severity, this federal law requires the performance of a biopsychosocial assessment by a multiprofessional team. This assessment must consider physical and functional impairments, contextual factors, performance limitations, and restrictions on social participation, in accordance with the parameters established by Law No. 13,146/2015, known as the Brazilian Inclusion Law of Persons with Disabilities.

### MONOCULAR VISION

At the federal level, Law No. 14,126/2021 became widely known for classifying monocular vision as a sensory disability [^8^]. However, published on the same date, Decree No. 10,654/2021 received less public attention [^9^]. This decree regulates the implementation of the law and establishes the requirement of a biopsychosocial assessment for the recognition of disability. In other words, the presence of monocular vision, by itself, does not necessarily imply the automatic recognition of disability status, despite the interpretation frequently disseminated in common understanding and in many administrative analyses of benefit concessions.

Overall, the loss of one eye causes a relevant functional impact during the initial adaptation period. However, over time, this condition tends not to prevent the performance of everyday activities. Although there is a reduction in visual field and depth perception, the healthy eye usually partially compensates for these limitations. Individuals with monocular vision often develop spontaneous adaptations, such as increased head movement and the use of indirect visual cues (e.g., shadows and relative sizes) to estimate depth [^10^].

These adaptive mechanisms make it possible to maintain a virtually normal routine, without the need for assistive technologies or specific accessibility resources. Such resources are described in Art. 2 of the CRPD:

“Communication” includes languages, the display of text, Braille, tactile communication, large print, accessible multimedia devices, as well as plain language, written and oral language, auditory systems, and digitized voice, and augmentative and alternative modes, means, and formats of communication, including accessible information and communication technology [^11^].

In this context, particular attention should be given to Public Civil Action No. 2009.51.01.026572-8, currently under review by the 8th Federal Court of the Judicial Section of Rio de Janeiro, filed by the Brazilian Institute for the Rights of Persons with Disabilities against the Federal Government, the State of Rio de Janeiro, and the Municipality of Rio de Janeiro. The action sought to prevent the appointment of individuals with monocular vision to positions reserved for PwDs, on the grounds that such classification would be inconsistent with the criteria established in Decree No. 3,298/1999. Subsequently, the Association of the Visually Impaired of the State of Rio de Janeiro requested its inclusion in the proceedings as an active co-litigant [^12^].

Within the scope of this case, the technical opinion signed by the ophthalmologists Dr. Newton Kara-José and Dr. Maria de Lourdes Veronese is particularly illuminating:

Based on the International Classification of Impairments, Disabilities, and Handicaps of WHO, an individual who presents blindness in one eye and normal vision in the other has “functional” vision, that is, participates in professional and social activities. Their “disability” is minimal, only related to activities that require binocular vision, such as operating a forklift or being an airplane pilot. There are no limitations in the execution of activities of daily living and there are no restrictions on social participation [^12^].

In light of this, the concern expressed by the institutions involved was that the automatic classification of monocular vision as a disability could compromise the principle of substantive equality. This principle presupposes unequal treatment of unequals according to the measure of their inequalities, with the objective of promoting equity of opportunity. The indiscriminate expansion of this classification could grant privileged treatment to individuals without significant functional disadvantage, distorting the logic of affirmative actions and potentially aggravating the exclusion of PwDs who effectively require assistive technologies or environmental adaptations for their social and professional inclusion [^13^].

The effects could be similar in the private sector. The so-called Quota Law for Persons with Disabilities (Art. 93 of Law No. 8,213/1991) establishes that companies with 100 or more employees must reserve between 2% and 5% of their positions for persons with disabilities or rehabilitated beneficiaries [^14^]. In this scenario, when faced with a choice between a candidate with severe visual impairment and another with monocular vision, it is plausible that the employer would choose the latter, both for operational and economic reasons. Although the monocular individual is classified as having “blindness” in one eye, according to the ICD-10 designation (H54.4), they generally do not require workplace adaptations or specific support resources.

By way of illustration, one may mention the case of a candidate for a public service examination with keratoconus, an ophthalmological disease that, in isolation, is not recognized as a disability but may cause significant impairments to activities and social participation [^15^]. This candidate presented the following visual acuity parameters:

a) without correction: 20/400 in both eyes;
b) with correction using glasses: 20/40 in the oculus dexter (OD) and 20/50 in the oculus sinister (OS);
c) with scleral lenses: 20/60 (OD) and 20/80 (OS).

His routine was marked by ocular irritation and frequent dryness, requiring constant use of lubricating eye drops. Corneal aberrations, associated with previous refractive surgery and with keratoconus itself, resulted in blurred vision, distortion halos, photophobia, difficulty in low-light environments, and reduced visual field, making adaptation to glasses difficult. Although scleral lenses represented a less unsatisfactory alternative, they could not be used for prolonged periods, requiring frequent removals throughout the day. It was therefore a significantly dysfunctional condition, incompatible with the “best optical correction” provided for in the legal criteria.

**Figure 1:**
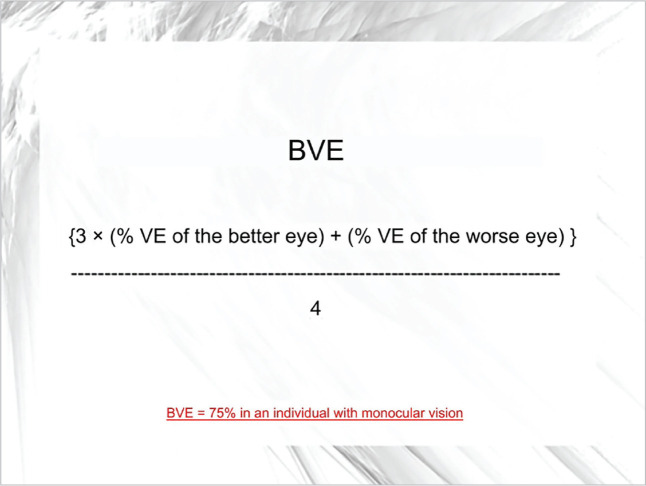
Calculation of BVE according to Braga et al. [^16^].

**Figure 2:**
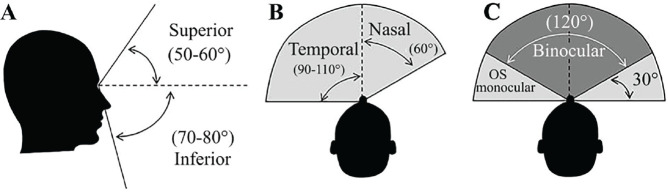
Visual fields according to Crossman & Neary [^17^]. A) vertical visual field; B) monocular horizontal visual field; C) binocular visual field.

Even if adaptation to the lenses were satisfactory, his visual capacity would remain substantially compromised, as demonstrated by the calculation of binocular visual equivalence (BVE), which assigns weight 3 to the eye with better acuity and weight 1 to the eye with worse performance, as illustrated in Figures 1 and 2:

BVE: binocular visual efficiency (VE).

OS: oculus sinister (left eye).

In the case described, the candidate presented an approximate BVE of 40%, whereas an individual with typical monocular vision presents about 75%. Paradoxically, the latter is recognized as a PwD for the purposes of reserving positions in public service examinations, while the former is not considered eligible for such classification, despite presenting more significant overall visual impairment.

**Figure 3:**
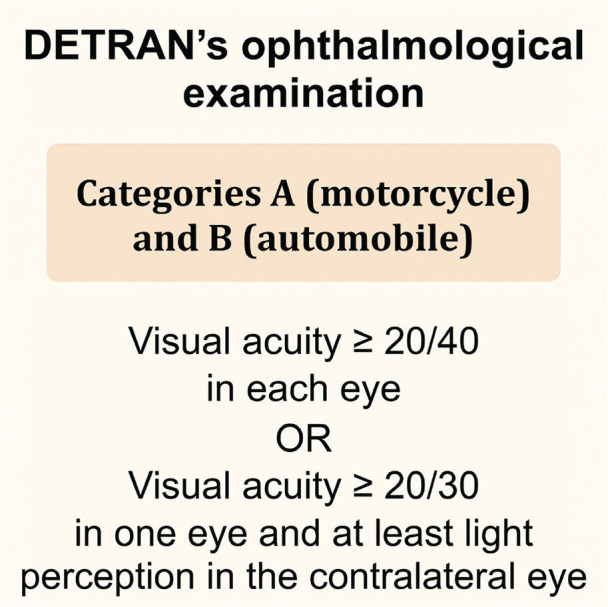
Visual acuity requirements for a driver’s license in categories A and B [^18^].

Finally, most of the more than 100 thousand Brazilians with monocular vision, frequently recognized as PwDs, remain eligible to obtain a National Driver’s License in categories A (motorcycle) and B (automobile), and may even perform professional activities such as taxi driving. In contrast, the candidate previously described did not meet the visual acuity requirements required for the renewal of his driver’s license (Figure 3), and had even been advised by medical recommendation to refrain from driving vehicles.

DETRAN: State Department of Traffic.

### AUTISM SPECTRUM DISORDER

Since the initial description made by Leo Kanner in 1943, focused on children with severe communication difficulties and restrictive behavioral patterns, the concept of ASD has undergone significant expansion. With the incorporation of conditions such as Asperger syndrome, characterized by preserved language and average or above-average intelligence, and with the adoption of the term spectrum in the Diagnostic and Statistical Manual of Mental Disorders, 5th edition (DSM-5), published in 2013, the diagnosis came to encompass a broad diversity of clinical manifestations.

Over the last 3 decades, a substantial increase in the number of ASD diagnoses has been observed. According to Allen Frances, the psychiatrist responsible for coordinating the development of the DSM-IV, this growth mainly results from four central factors [^19^]:

a) greater vigilance and identification by physicians, teachers, family members, and by patients themselves;
b) social pressure for greater access to therapeutic and educational services;
c) introduction of the DSM-IV (1994), which expanded the concept of ASD by incorporating Asperger disorder, a condition with imprecise boundaries in relation to socially eccentric or less adapted individuals;
d) reduction of stigma associated with the diagnosis. In this context, the internet facilitated more comfortable forms of communication, expanded interpersonal support, and fostered a sense of belonging among individuals with similar characteristics. At the same time, the disorder began to receive broad media coverage, and some public figures came to recognize themselves in the definition of Asperger syndrome and adopted it publicly. In certain professional environments, particularly in fields such as computing and information technology, the diagnosis even began to acquire a certain symbolic value.

According to Frances, psychiatric classifications, when poorly understood, exert strong influence on prevalence estimates, which are highly sensitive to changes in diagnostic criteria. The author himself acknowledges:

As leader of the DSM-IV task force, I deserve the blame for not anticipating the runaway overdiagnosis of Asperger disorder. It would have been helpful to foresee the change in diagnostic rates and explain its causes. We should have taken proactive measures to educate the public and the media about what the labels mean, and emphasized that the change did not occur in the children, but in the way they are diagnosed. It is much easier to start a fad than to stop it [^19^].

In other words, the increase in diagnoses largely reflects the expansion of access to clinical assessments, the redefinition of diagnostic criteria, and the emergence of a new perception of ASD as an identity. People with ASD, especially those classified as high functioning, have sought recognition, inclusion, and rights, fostering activism that values neurodiversity and often questions exclusively medical approaches to the phenomenon [^20^].

The classification of mental disorders became even more flexible with the publication of the DSM-5 in 2013 [^21^]. Although the manual was widely celebrated outside academic circles, it received consistent criticism from specialists, particularly due to the growing medicalization of everyday behaviors and the so-called “diagnostic inflation,” i.e., the tendency to pathologize normal variations of human experience. Criticism was also raised regarding the transparency of the manual’s development process [^22^,^23^].

As ASD constitutes a spectrum with heterogeneous functional repercussions, the automatic characterization of the disorder as a disability requires careful analysis. Particular attention should be given to diagnoses made only in adulthood. In many of these cases, individuals have developed adaptive strategies over time, building diverse and, not rarely, successful personal and professional trajectories. This observation does not exclude the possibility of psychological distress, underreporting, or historical invisibility, but indicates that such situations do not always fit within traditional models of disability.

This concern is also reflected in recent legal debates. A case currently under review in the Federal Justice system seeks to determine whether an ASD diagnosis exempts the need for a biopsychosocial assessment for the characterization of disability status in the context of access to the Continuous Cash Benefit (BPC, in Portuguese). Regarding this judgment, the National Association of Members of the Public Prosecutor’s Office for the Defense of the Rights of Older Persons and Persons with Disabilities published a clarification note, from which the following points stand out:

Although the waiver of the biopsychosocial assessment may be interpreted by some as an advance in guaranteeing rights for autistic individuals, this measure actually represents a setback in the process of implementing the social model of disability, as it reinforces the medical model of disability. This model is based exclusively on clinical diagnoses, without considering the social, environmental, and individual barriers that affect a person’s participation in society. Such a perspective contradicts the principles established by the Convention on the Rights of Persons with Disabilities (CRPD), an international treaty adopted by the United Nations (UN) and ratified by Brazil with constitutional amendment status.

Furthermore, admitting that diagnosis alone is sufficient to characterize disability for the purposes of access to the Continuous Cash Benefit (BPC) will open a precedent for other diagnoses eventually recognized by legislation to receive the same treatment. Currently, approximately 60 bills are under consideration in the Chamber of Deputies proposing the recognition of different diagnoses as disabilities, including fibromyalgia, chronic kidney disease, alopecia areata, diabetes mellitus, neurofibromatosis, cleft palate, systemic lupus, sickle cell anemia, Tourette syndrome, keratoconus, Crohn’s disease, and other conditions. Indeed, if the understanding of the National Panel for the Standardization of Federal Case Law (TNU) is established in the sense of waiving the biopsychosocial assessment for ASD, it may encourage the expansion of this reasoning to several other health conditions, disregarding the analysis-mandatory in the social model of disability-of the real barriers that impact the social participation of these individuals.

Therefore, the TNU’s decision regarding Theme 376 will have implications that go beyond access to the BPC. Its unfolding may affect the entire conception of disability in Brazil, influencing future legal and legislative interpretations. Moreover, it may compromise the adoption of the biopsychosocial model, which constitutes the international and national normative basis for the protection and inclusion of persons with disabilities [^3^].

In a number of countries (e.g., United States, United Kingdom, Australia, Chile, Argentina, and Spain), an ASD diagnosis does not automatically ensure access to social benefits. In such systems, a careful assessment of the functional impacts of the disorder on the individual’s life is required [^24^].

Finally, Judy Singer, responsible for introducing the concept of neurodiversity, later began to criticize the distortion of her original proposal. According to the author, her initial reflections referred mainly to individuals with high-functioning ASD or Asperger syndrome, whose reality cannot be equated with that of individuals with classic ASD. The indiscriminate inclusion of all cases within a single group would hinder the recognition of the different needs that exist among individuals, regardless of whether they identify themselves as PwDs.

Singer herself began to argue that the concept of neurodiversity was progressively transformed into an excessively optimistic ideological perspective, which she compared to a “Pollyanna/Pangloss” view. In contrast, she proposed the concept of neurorealism, which emphasizes the need to concretely recognize the cognitive and neurological limitations present in certain clinical conditions [^25^,^26^].

## CONCLUSIONS

The conception of disability is currently undergoing a process of transition, shifting from a strictly pathological perspective to a broader biopsychosocial approach. This transformation is aligned with the international disability model adopted by Brazil and enshrined in international treaties.

The emergence of legal provisions that define the condition of PwD exclusively on the basis of medical diagnoses raises concern, as they disregard the multiple factors that influence functioning and social participation.

It is possible that such legislative initiatives originate from well-intentioned social movements. However, they may reflect sectoral demands that do not always correspond to the collective interest. As noted by Yuval Harari, historian, philosopher, and bestselling author:

Even if you belong to a disadvantaged group and have firsthand understanding of its perspectives, this does not mean that you understand the perspectives of all other similar groups. For each group and subgroup faces different entanglements of vulnerabilities, unequal treatment, coded insults, and institutional discrimination.

All existing human tribes are committed to advancing their particular interests rather than understanding the global truth [^27^].

This type of approach, potentially reductionist, may increase inequalities among different groups of PwDs, compromising social inclusion and hindering the equitable distribution of rights and benefits.

This highlights the need for normative harmonization, especially in light of the persistent limitation of public resources. Although the demands of certain social groups are legitimate, it is the responsibility of the State to establish prudent and reasonable criteria for defining priorities, directing resources toward individuals who face greater barriers to social participation.

In this regard, the Modified Brazilian Functionality Instrument is currently under development, proposed by the Federal Government as a unified tool for disability assessment. The instrument incorporates the principles of the ICF and promotes an integrated approach that considers biological, psychological, and social dimensions [^28^,^29^].

In view of this scenario, it becomes appropriate for scientific and professional institutions to take a critical position regarding the characterization of disability based exclusively on nosographic criteria, an approach already considered outdated in light of the principles of the ICF, widely disseminated by the WHO.

This misguided view does not necessarily reflect what is best for the collective, but rather sectional interests supported by legislators who may be more concerned with not displeasing their electoral bases.

This debate, often labeled politically sensitive, must be conducted transparently and responsibly, reconciling the ideal of equity with the State’s duty of public accountability, in order to ensure the most efficient possible allocation of resources that are known to be scarce [^30^].

## Data Availability

The data supporting the findings of this study are available within the article.

## References

[1] Brasil (1999). Regulamenta a Lei nº 7.853, de 24 de outubro de 1989, dispõe
sobre a Política Nacional para a Integração da Pessoa
Portadora de Deficiência, e dá outras
providências.

[2] Brasil (2015). Institui a Lei Brasileira de Inclusão da Pessoa com
Deficiência (Estatuto da Pessoa com Deficiência).

[3] Associação Nacional dos Membros do Ministério
Público de Defesa dos Direitos das Pessoas Idosas e Pessoas com
Deficiência (AMPID) (2025). Nota sobre o tema 376 da Turma Nacional de Uniformização
dos Juizados Especiais Federais (TNU) 2025.

[4] Goldenberg DL., Connor RF (2025). Fibromyalgia: Clinical manifestations and diagnosis in adults.

[5] Papadakis MA, McQuaid KR, Rabow MC. (2025). Current Medical Diagnosis & Treatment.

[6] Kamiński M, Hrycaj P. (2024). Celebrities influence on rheumatic diseases interest: A Google
Trends analysis. Rheumatol Int.

[7] Brasil (2025). Lei nº 15.176, de 23 de julho de 2025.

[8] Brasil (2021). Classifica a visão monocular como deficiência sensorial,
do tipo visual.

[9] Brasil (2021). Decreto nº 10.654, de 22 de março de 2021.

[10] Silva MRD, Nobre MIRS, Carvalho KMD, Montilha RDCI. (2014). Visual impairment, rehabilitation and international
classification of functioning, disability and health. Rev Bras Oftalmol.

[11] Brasil (2009). Decreto nº 6.949, de 25 de agosto de 2009. Promulga a Convenção Internacional sobre os Direitos das
Pessoas com Deficiência e seu Protocolo Facultativo, assinados em
Nova York, em 30 de março de 2007.

[12] Freitas LCDSR. (2010). Visão monocular não é deficiência.

[13] Souza RC, Nogueira JNT, Dourado ACA, Medeiros Félix G, Silva Martins VH. (2024). Visão monocular: avaliação da pessoa com
deficiência em benefícios assistenciais
judicializados. Cad Pedag.

[14] Matos HDNF, Raiol RWG. (2018). Pessoas com deficiência e seu direito à
inclusão no mercado de trabalho. Rev Eletron Curso Direito UFSM.

[15] Dudeja L, Chauhan T, Vohra S. (2021). Sequence of events leading to diagnosis of keratoconus and its
impact on quality of life. Indian J Ophthalmol.

[16] Braga BE, Santos IC, Rodrigues S, Nakano SMS. (2012). Perícia médica.

[17] Crossman AR, Neary D. (2014). Neuroanatomy: an illustrated colour text.

[18] Brasil, Conselho Nacional de Trânsito (CONTRAN) (2022). Resolução nº 927, de 28 de novembro de 2022.

[19] Frances A. (2017). Voltando ao normal: como o excesso de diagnósticos e a
medicalização da vida estão acabando com a nossa
sanidade e o que pode ser feito para retomarmos o controle.

[20] Bialer M, Voltolini R. (2022). Autismo: história de um quadro e o quadro de uma
história. Psicol Estud.

[21] Zorzanelli R, Bezerra B, Costa JF, Bezerra B Jr (2014). A criação de diagnósticos na psiquiatria
contemporânea.

[22] Lieberman J, Ogas O. (2016). Psiquiatria: uma história não contada.

[23] Pondé MP. (2018). A crise do diagnóstico em psiquiatria e os manuais
diagnósticos. Rev Latinoam Psicopatol Fundam.

[24] Bernardes LCG. (2025). Autismo e deficiência: a avaliação biopsicossocial
é necessária?.

[25] Lage A. (2006). Movimento diz que autismo não é
doença. Folha Online.

[26] Lutz A. (2023). An interview with neurodiversity originator Judy
Singer. Psychol Today.

[27] Harari YN. (2018). 21 lições para o século 21.

[28] Nunes LCA, Leite LP, Amaral GFD. (2022). Análise do Índice de Funcionalidade Brasileiro Modificado
(IFBr-M) e suas implicações sociais. Rev Bras Educ Espec.

[29] Coelho ACF, Petersen RDS, Köptche LS, Morais IA, Augusto NNO, Cardoso HE (2024). Produto técnico 03: manual de aplicação do
instrumento de funcionalidade brasileiro - IFBrM.

[30] Nunes R. (2017). Ensaios em bioética.

